# RNA sequencing identifies transcriptional changes in the rabbit larynx in response to low humidity challenge

**DOI:** 10.1186/s12864-020-07301-7

**Published:** 2020-12-11

**Authors:** Taylor W. Bailey, Andrea Pires dos Santos, Naila Cannes do Nascimento, Shaojun Xie, Jyothi Thimmapuram, M. Preeti Sivasankar, Abigail Cox

**Affiliations:** 1 Department of Comparative Pathobiology, Purdue University, West Lafayette, IN 47907 USA; 2 Department of Public Health, Purdue University, West Lafayette, IN 47907 USA; 3 Department of Speech, Language, and Hearing Sciences, Purdue University, West Lafayette, IN 47907 USA; 4 Bioinformatics Core, Purdue University, West Lafayette, Indiana, 47907 USA

**Keywords:** Animal model, In vivo, Vocal folds, Airway, Dehydration, RNA-Seq

## Abstract

**Background:**

Voice disorders are a worldwide problem impacting human health, particularly for occupational voice users. Avoidance of surface dehydration is commonly prescribed as a protective factor against the development of dysphonia. The available literature inconclusively supports this practice and a biological mechanism for how surface dehydration of the laryngeal tissue affects voice has not been described. In this study, we used an in vivo male New Zealand white rabbit model to elucidate biological changes based on gene expression within the vocal folds from surface dehydration. Surface dehydration was induced by exposure to low humidity air (18.6% + 4.3%) for 8 h. Exposure to moderate humidity (43.0% + 4.3%) served as the control condition. Ilumina-based RNA sequencing was performed and used for transcriptome analysis with validation by RT-qPCR.

**Results:**

There were 103 statistically significant differentially expressed genes identified through Cuffdiff with 61 genes meeting significance by both false discovery rate and fold change. Functional annotation enrichment and predicted protein interaction mapping showed enrichment of various loci, including cellular stress and inflammatory response, ciliary function, and keratinocyte development. Eight genes were selected for RT-qPCR validation. Matrix metalloproteinase 12 (*MMP12*) and macrophage cationic peptide 1 (*MCP1*) were significantly upregulated and an epithelial chloride channel protein (*ECCP)* was significantly downregulated after surface dehydration by RNA-Seq and RT-qPCR. Suprabasin (*SPBN*) and zinc activated cationic channel (*ZACN*) were marginally, but non-significantly down- and upregulated as evidenced by RT-qPCR, respectively.

**Conclusions:**

The data together support the notion that surface dehydration induces physiological changes in the vocal folds and justifies targeted analysis to further explore the underlying biology of compensatory fluid/ion flux and inflammatory mediators in response to airway surface dehydration.

## Background

Voice disorders are a prevalent communication disorder affecting human health worldwide [1–6]. In the United States general population, the prevalence of voice disorders has been estimated at 6.2% [7], and more recently, at 7.6% [8]. Data from the National Longitudinal Study of Adolescent to Adult Health shows the same 6% estimate among the adolescent population [9]. The development of voice disorders is identified as an occupational hazard, particularly among speakers who depend on a healthy voice for their livelihood. School teachers, entertainers, legal professionals are all at greater risk of dysphonia from voice disorders [3, 7, 10–13]. The economic impact of voice disorders is substantial. The average associated health care costs in the United States have been estimated at almost 200 million dollars [14], and a study of Brazilian teachers having to take time away from work due to dysphonia illustrates the potential impact of a loss of productivity in the workforce [15]. Taken together, the impact of voice disorders on society supports the need for a more comprehensive understanding of the development of voice disorders and therapies to address them.

Interventions for voice disorders exist along a continuum of non-invasive behavioral modifications to phonosurgery. The focus of this study is on the molecular biological responses to laryngeal surface dehydration as a means of substantiating the commonly prescribed prophylactic and therapeutic practice among speech-language pathologists [4, 16–19].

Dehydration, as it relates to voice, occurs under two paradigms: systemic dehydration and airway surface dehydration. Systemic dehydration, decreased total body water, has been shown to negatively impact phonatory effort in humans and acoustic measures in humans and ex vivo animal models [20–23]. Surface dehydration as related to voice is defined as loss of water from the luminal surface of the larynx and vocal folds. In everyday life, this may be caused by exposure to air of low humidity or increased respiratory rate from exercise. While there is evidence suggesting that surface dehydration within the larynx negatively impacts phonation with similar outcomes as systemic dehydration, recent studies in humans [24–27] do not always find a significant correlation between the two.

Unfortunately, rigorous in vivo analysis of the physiology of laryngeal surface dehydration is precluded by the invasive nature of data collection and the ethical implications of causing vocal injury in human subjects. Human studies are, therefore, generally limited to acoustic and aerodynamic measures or post-mortem evaluation. Conversely, animal models have largely allowed for ex vivo studies, which provide ample evidence that surface dehydration impacts vocal fold biomechanics and function [28–30], but the molecular pathobiology and resulting homeostatic compensatory mechanisms remain unclear. An attractive surrogate to the vocal folds is the airway distal to the larynx, which has been studied in the context of airway surface fluid homeostasis and response to luminal perturbations [31–33]. It has long been established that the humidity of inspired air can affect the magnitude of water lost to respiration [34] and that the resulting concentration of luminal electrolytes can cause dramatic physiological responses in the trachea, upper and lower airways [35, 36]. The vocal folds are covered by nonkeratinized stratified squamous epithelium, and the laryngeal lumen is predominately covered by respiratory epithelium. Therefore, the larynx may respond to perturbations similarly to the tracheal epithelium. This potential is supported in studies assessing vocal fold ion flux to altered composition of luminal surface fluid [37–39]. However, these were in vitro studies limiting the generalization of the data. Further studies are required to address questions of the specific underlying biology.

To probe for potential physiological responses to surface dehydration, we used an in vivo rabbit model. Anatomically, the rabbit larynx is grossly similar to the human larynx. Its size has been approximated to 8.6 × 5.5 mm at the level of the arytenoids [40, 41], consistent with the dimensions of the human newborn larynx [42]. Additionally, the literature demonstrates that rabbit larynges exhibit sufficient biological similarity to humans and have been used in molecular and histological studies of the vocal folds [43–47]. The rabbit larynx has also been used to characterize the physiological response to injury secondary to phonation [46, 47] or laryngeal and vocal fold surgery [48–50]. The common use of rabbits for laryngeal studies and the relatively small size for handling and housing makes this animal a suitable model for this study.

In this study, we sought to identify transcriptional-level changes in response to low humidity exposure that suggest a response to surface dehydration within the membranous vocal folds or the vocal fold lamina propria. We successfully addressed the following aims: [1] construction and evaluation of an environmental chamber capable of exposing rabbits to a consistent, physiologically-realistic low relative humidity environment and [2] investigation of the effects of 8 h of low humidity exposure on rabbit larynx by way of RNA sequencing (RNA-Seq). An 8-h exposure was selected as representative of a typical working day for human subjects. We used low humidity rather than desiccated air as the surface dehydration challenge to increase the ecological validity of the study. Rabbits exposed to moderate humidity served as the control condition.

## Results

### Humidity challenge and gross physical assessment

**Fig. 1:**
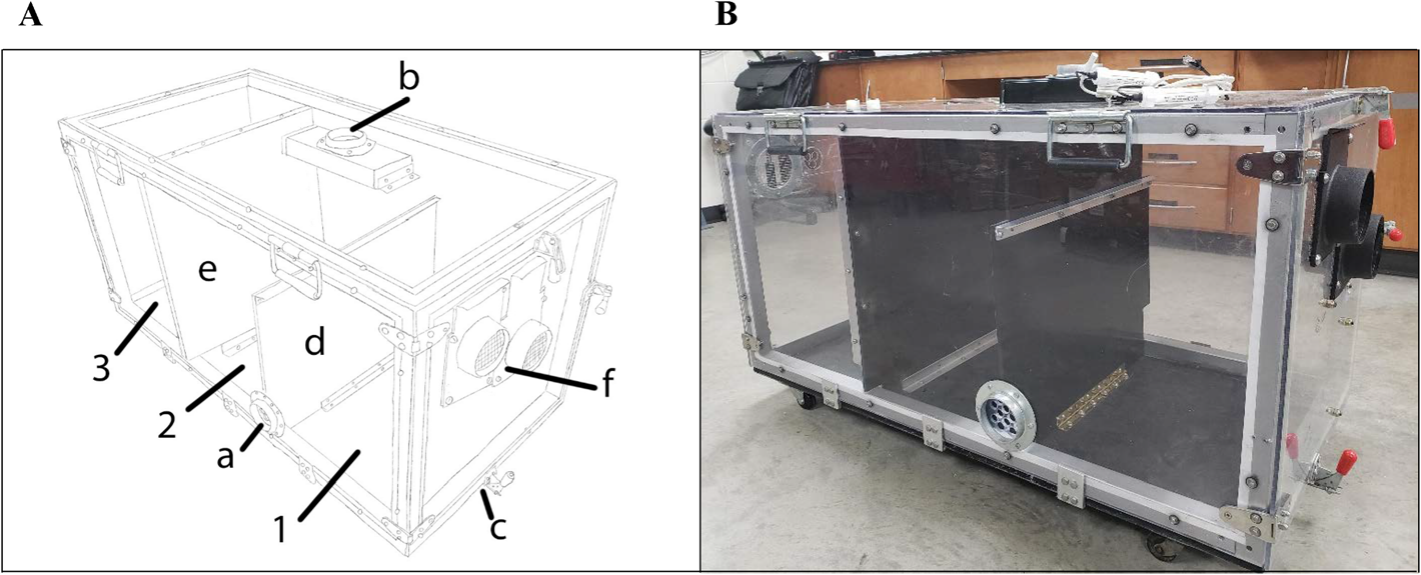
Environmental chamber used in this experiment. **a** Schematic design of the environmental chamber. Air output toward dehumidifier (a), air intake plenum from dehumidifier (b), latches for chamber doors that open longitudinally (c), mobile divider for separating challenge compartment into two sections (d, 1, 2), permanent divider separating challenge from control compartment (e, 3), and gated vent caps for titration of room air (f). **b** Picture of chamber showing actual materials and dimensions. Schematic design and photograph are both property of the authors

**Fig. 2:**
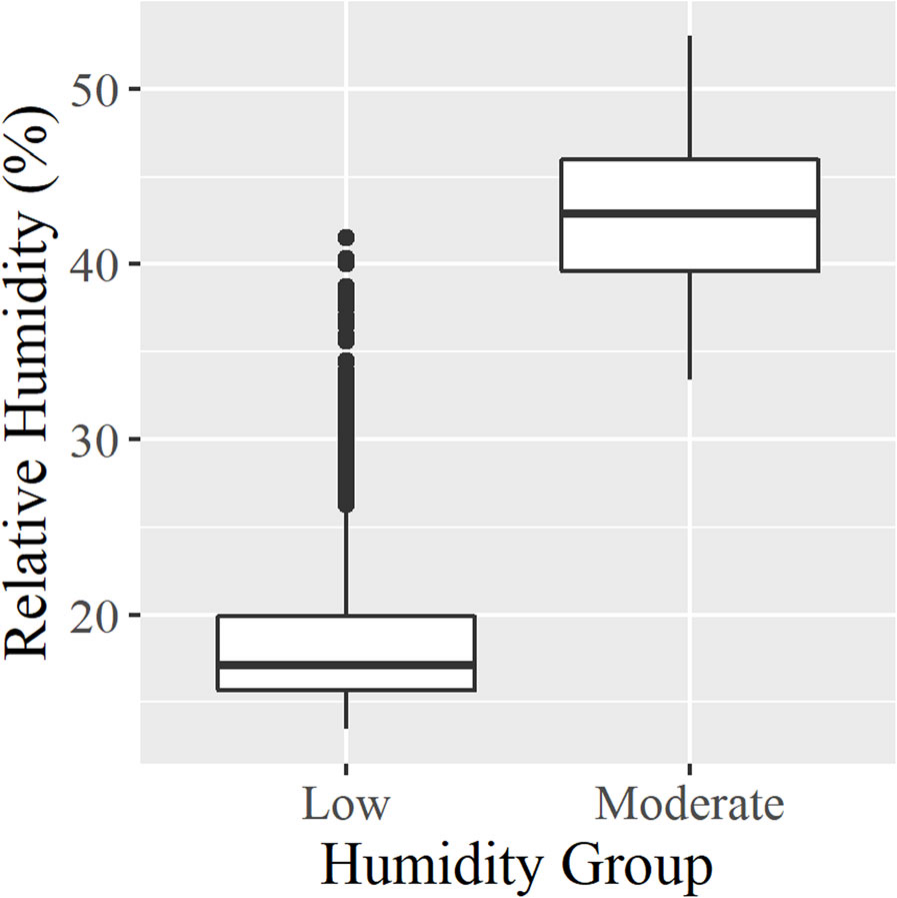
Relative humidity measured during experimental exposures of 8 h. Aggregate data for relative humidity measured across all experiments for each group. Box plots represent the quartiles of the population distribution

A total of eight rabbits were challenged with low humidity, and six rabbits were exposed to moderate humidity (control condition) in a specially fabricated environmental chamber (Fig. 1; see Methods section for details). Low humidity was 18.6 ± 4.3% (mean ± standard deviation) over the 8 h. The moderate humidity exposure was 43.0 ± 4.3% over the 8 h (Fig. 2). There was no observable behavioral differences or evidence of respiratory distress following exposure in either group. No gross evidence of inflammation or damage to the laryngeal mucosa was observed during visual examination under a dissecting microscope.

### Packed cell volume (PCV)

The pre-experiment PCV (%) across all 14 rabbits was 46.7 ± 2.8 (mean ± SD). The % change in PCV from baseline to after the experiment did not differ significantly between the low and moderate humidity groups (*p* = 0.1692).

### Sequence read mapping and RNA-Seq

Approximately 69 to 112 million paired reads were obtained by RNA-Seq with an average of 70% quality reads mapping to genes in the rabbit genome in each sample. In total, 23,669 annotations were obtained. Differential gene expression by Cuffdiff revealed 103 genes reaching an FDR < 0.05 with 61 meeting the additional fold change (|log2 FC| ≥ 1) filtering criterion. Of these, 48 genes were considered significantly downregulated and 13 genes were significantly upregulated. The 10 genes with the greatest up- and downregulated fold changes from this list of 61 are shown in Table 1. A complete list of all genes identified is provided within the Additional file 1: Table S1.

**Table 1:** List of the ten most significantly upregulated and downregulated genes as identified by RNA-Seq

ENSEMBL ID	Gene symbol	log2 FC	FDR	Biomart Annotation
ENSOCUG00000003548	*ECCP* ^*a*^	−2.574	0.0121	epithelial chloride channel protein^b^
ENSOCUG00000024036	*COL6A5*	−2.550	0.0121	collagen type VI alpha 5 chain
ENSOCUG00000013994	*PLA2G4D*	−2.529	0.0121	phospholipase A2 group IVD
ENSOCUG00000010912	*KRTDAP*	−2.505	0.0121	keratinocyte differentiation associated protein
ENSOCUG00000011842	*CRNN*	−2.197	0.0121	cornulin
ENSOCUG00000029191	*–*	−2.072	0.0121	Immunoglobulin lambda variable precursor^c^
ENSOCUG00000011037	*MYH7*	−2.030	0.0121	myosin heavy chain 7
ENSOCUG00000014187	*MINAR1*	−2.009	0.0212	membrane integral NOTCH2 associated receptor 1
ENSOCUG00000008772	*FANK1*	−1.905	0.0121	fibronectin type III and ankyrin repeat domains 1
ENSOCUG00000011472	*FOXJ1*	−1.854	0.0121	forkhead box J1
ENSOCUG00000013331	*–*	1.469	0.0121	glutathione peroxidase^c^
ENSOCUG00000027549	*–*	1.497	0.0212	immunoglobulin heavy constant IG chain C^c^
ENSOCUG00000016426	*AGER*	1.516	0.0300	advanced glycosylation end-product specific receptor
ENSOCUG00000006499	*MGARP*	1.566	0.0121	mitochondria localized glutamic acid-rich protein
ENSOCUG00000007106	*RAE2*	1.689	0.0121	ribonuclease 8
ENSOCUG00000027406	*LDHA*	1.747	0.0120	lactate dehydrogenase A chain^c^
ENSOCUG00000024691	*ATPB*	1.856	0.0121	ATP synthase subunit B^c^
ENSOCUG00000024788		1.941	0.0121	L-lactate dehydrogenase A chain-like
ENSOCUG00000003229	*MCP-1*	2.226	0.0121	macrophage cationic peptide 1^b^
ENSOCUG00000008303	*MMP12*	2.277	0.0364	matrix metallopeptidase 12^b^

The twenty genes listed meet both filtering criteria of FDR < 0.05 and |log2 FC| ≥ 1. Annotations were obtained with Biomart from references to NCBI database information

^a^*ECCP* is not a formal gene symbol and is used for the purpose of this study

^b^Genes selected for validation by RT-qPCR

^c^Annotation not available through Biomart and was obtained by a search of ENSEMBL database by ID. Negative and positive values of log2 FC denote down- and upregulated genes, respectively

**Fig. 3:**
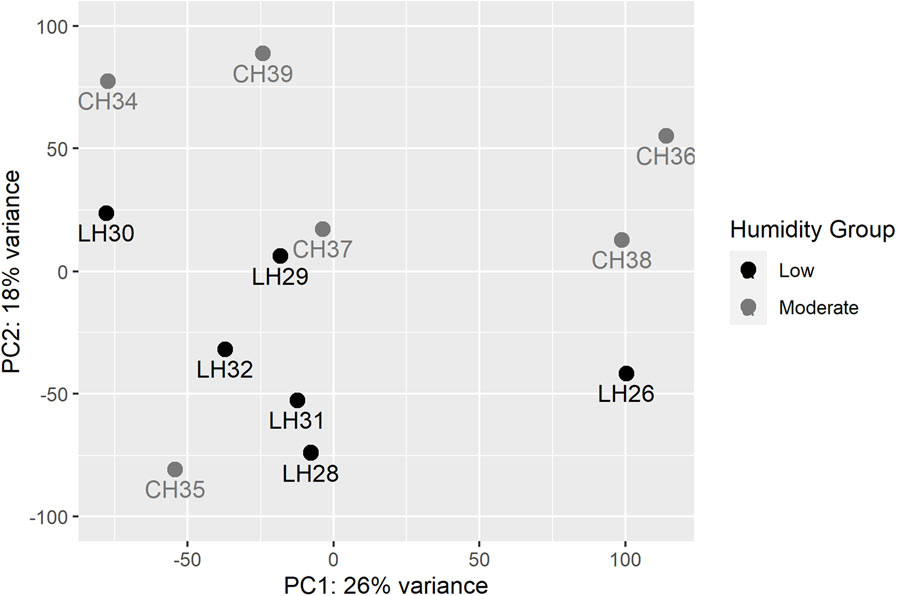
Principal component analysis of rabbits across groups based on FPKM obtained by Cuffdiff

Rabbits were compared using principal component analysis based on FPKM obtained from Cuffdiff without low expression genes being removed (Fig. 3). The first two principal components explain 44% of the total variability. Although neither PC1 nor PC2 were able to distinguish low humidity rabbits from control rabbits, rabbits tended to cluster according to their treatment information based on PC1 and PC2 together. The rabbits with the most prominent deviations, LH26 and CH35, were not found to be consistent outliers within the qRT-PCR analyses discussed below.

### Functional enrichment analysis

Functional enrichment analysis by DAVID and STRING provided similar but distinct sets with FDR < 0.05. DAVID identified 4 GO terms for biological process, 6 GO terms for cellular component, 2 GO terms for molecular function, and 7 processes by KEGG with FDR < 0.05. GO terms and KEGG processes included cardiac muscle function, calcium binding, chemical carcinogenesis, and ECM-receptor interaction. STRING provided a richer set with 7, 15, and 19 GO terms for biological process, cellular component, and molecular function, respectively, and 2 KEGG processes. GO terms included stress and inflammatory response, cytoskeleton, and ion binding.

For GSEA, 17 genes sets were significantly enriched in the moderate humidity group with an FDR < 0.25. There were 5, 6, 6 terms for biological process, cellular compartment, and molecular function, respectively. These include collagen, basement and plasma membrane, epidermis development, and epithelial cell differentiation. In the low humidity group 4 gene sets were significantly enriched with FDR < 0.25. There were 2, 1, and 1 terms for biological process, cellular compartment, and molecular function, respectively. These include olfactory receptor activity and cellular response to calcium. The full lists of terms, functions, associated genes, and statistics for the aforementioned DAVID and STRING analyses, and enrichment data in moderate and low humidity groups from GSEA are provided in Additional file 2: Table S2.

**Fig. 4:**
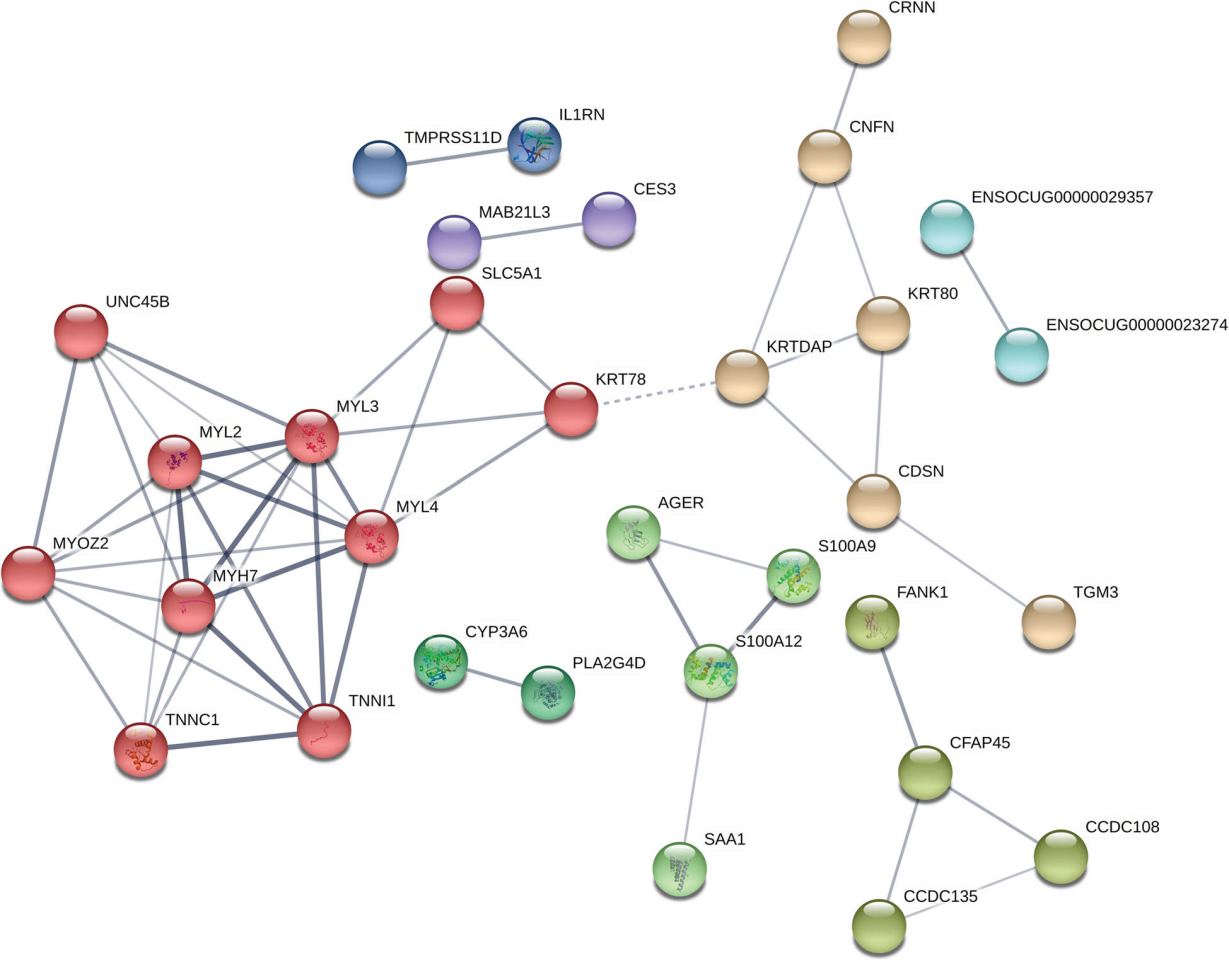
Protein interaction network was created using STRING. A 100 node network was obtained from an input set of 103 differentially expressed genes identified by Cuffdiff with an FDR < 0.05. The line thickness represents the strength of the data to support the interaction, including text mining, experimental, database, co-expression, neighborhood, gene fusion, and co-occurrence sources. The minimum required interaction score was set to 0.4. Shell parameters were set to “None”. Disconnected nodes are not shown. Cluster colors are based on the Markov Cluster Algorithm with the inflation parameter set to 2

The predicted protein-interacting network generated by STRING is shown in Fig. 4. There were 8 clusters identified with between 2 to 10 gene products. Larger clusters contain members that are associated with cellular response to external stimuli and immune response (dark green, lavender), muscle function (red), keratinocyte development (light green), and ciliary function (aqua).

### RT-qPCR validation

Eight genes were selected for subsequent data validation by RT-qPCR based on their predicted functions and assumption of relevance to vocal fold or laryngeal physiology; they consist of ENSOCUG00000003548, annotated as an epithelial chloride channel protein which will be referred to as “*ECCP*”, cadherin related family member 4 (*CDHR4*), corneodesmosin (*CDSN*), macrophage cationic peptide 1 (*MCP1*), matrix metallopeptidase 12 (*MMP12*), suprabasin (*SPBN*), zinc activated cationic channel (*ZACN*), and mucin 21 (*MUC21*), although the absolute value of log2 FC for *MUC21* by RNA-Seq was only 0.79.

**Fig. 5:**
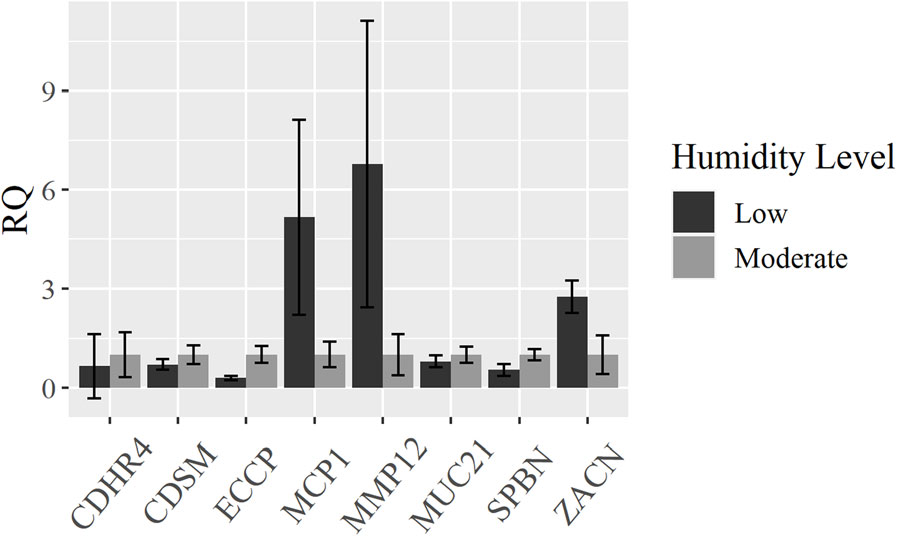
RT-qPCR validation. Relative quantification for each gene was determined by the ΔΔCt method. All reactions were run in triplicate. The level of expression of each tested gene was standardized to the housekeeping gene *HPRT1*, and ΔΔCt was calculated using the average of the ΔCts from the control group for the respective gene. *ECCP*, *MCP1* and *MMP12* were significantly different (*p* < 0.05) and *SPBN* and *ZACN* marginally non-significant (*p* = 0.06). Differences between groups as determined by the Welch t-test. Results represent 5–7 samples/group for each gene after the removal of outlier values as determined by the iterative application of a two-tailed Grubb’s test. Error bars represent the SEM for relative quantification within the respective humidity group

Of the eight genes tested, significant differences in relative expression were validated for *ECCP* (*p* = 0.028), *MCP1* (*p* = 0.030), and *MMP12* (*p* = 0.045) and were marginally non-significant for SPBN (*p* = 0.067) and *ZACN* (*p* = 0.066). The most prominent fold changes between the low and moderate humidity groups was observed for *MMP12* (FC = 6.8), *MCP1* (FC = 5.2), and *ZACN* (FC = 2.76). *ECCP* exhibited the largest downregulation (FC = 3.74). The remaining genes exhibited non-significant changes despite differential expression by RNA-Seq analysis (Fig. 5). Comparison of data from RNA-Seq and RT-qPCR are provided in Table 2.

**Table 2:** Summary of genes selected for follow up analysis by RT-qPCR

Ensembl ID NCBI Gene ID	Gene	log2 FC RNA-Seq	FDR RNA-Seq	log2 FC qPCR	*P*-value qPCR
ENSOCUG00000003548 100352679	*ECCP*^a^	−2.57	0.01	−1.796	0.028
ENSOCUG00000009174 100358424	*CDHR4*	−1.80	0.01	−0.618	0.363
ENSOCUG00000006280 100338321	*CDSN*	−1.32	0.01	−0.513	0.186
ENSOCUG00000003229 100009115	*MCP1*	2.23	0.01	2.371	0.030
ENSOCUG00000008303 100009559	*MMP12*	2.28	0.04	2.764	0.045
ENSOCUG00000001869 108177417	*MUC21*	−0.79	0.01	−0.329	0.228
ENSOCUG00000010917 100346157	*SPBN*	−1.15	0.01	−0.905	0.067
ENSOCUG00000000422 100358831	*ZACN*	1.27	0.01	1.466	0.066

^a^*ECCP* is not a formal gene symbol and is used for the purpose of this study

### In silico analysis of ENSOCUG00000003548 gene (*ECCP*)

ENSOCUG00000003548 maps to NCBI gene accession number 100352679, annotated as epithelial chloride channel protein. This gene lies downstream of LOC100338755 (calcium-activated chloride channel regulator 4-like), calcium-activated chloride channels 4, 2, and 1 (*CLCA4, CLCA2, CACL1*).

## Discussion

The transcriptional changes observed in this study indicate that just 8 h exposure to a low humidity environment can adversely affect vocal fold biology. To the best of our knowledge, this is the first study to demonstrate the effects of surface dehydration on vocal fold tissue in vivo. Important to our methodology, evaluation of the change in PCV following experimental challenge ruled out systemic dehydration as an unintended confounding factor in our analysis. There is considerable evidence that systemic dehydration negatively impacts phonation [20–23]. Surface dehydration represents a loss of water from the mucosal surface of the larynx, and while some level of local tissue water loss may be experienced through compensatory rehydration of the epithelial surface, we would not expect systemic dehydration to result. We hypothesize that the homeostatic responses to surface and systemic dehydration are governed by different cellular mechanisms, thus we used % PCV change to control for unintended systemic consequences of low humidity exposure with the concomitant withholding of food and water.

We developed a method to efficiently challenge rabbits to low humidity. We achieved average low relative humidity of approximately 20%, representing physiologically-realistic and substandard occupational conditions per Occupational Safety and Health Administration (OSHA) recommendations [51]. Moderate humidity control exposures were conducted in the same chamber with all compartments open to room air of variable temperature within housing guidelines for rabbits. Low humidity challenge and moderate humidity exposure could not be conducted at the same time because preliminary tests demonstrated that a fully closed air circuit that is needed to lower humidity in the chamber measurably increased the interior temperature of the compartments. By separating them, we successfully maintained appropriate ambient temperatures for the low humidity exposures [52] and maintained a 2-fold increase in moderate humidity exposures.

It is noteworthy that exposure to low relative humidity below the Occupational Health and Safety Administration (OHSA) recommended limit of 20% induced transcriptional changes within functional gene categories including inflammation, ion transport, and keratinocyte development. The most robust functional enrichments identified by STRING were stress, defense, and inflammatory responses. Additionally, outside of the STRING analysis, various genes for immunoglobulin chains were identified, three of which were downregulated and one that was upregulated. Interestingly, this cluster presents two opposing interpretations of innate immune dampening and possible macrophage activation.

While none of these genes or corresponding proteins are described within the larynx, the downregulated cluster can be interpreted as a dampening of acute inflammatory response. ORM1 and SAA1 are both acute phase proteins. ORM1 is an acute phase protein that has been shown to polarize M2 macrophage differentiation [53] and to enhance epithelial integrity in a culture model of the blood-brain barrier [54]. While ORM1 exhibits anti-inflammatory activity and its downregulation may allow for the development of a more robust inflammatory process, it may also be interpreted as indicative of surface dehydration not contributing to an activating inflammatory event. SAA1 is also an acute phase protein and is associated with a variety of pathological conditions, but it has also been shown to positively influence keratinocyte activity [55]. The S100 proteins are diverse with involvement in several cellular processes, but both S100A9 [56] and S100A12 [57] have been described as damage associated molecular patterns in the literature. Taken together, these results suggest that either surface dehydration is not inducing inflammatory pathways or that there is active repression of pro-inflammatory mediators. The latter is substantiated by the increase of *IL1RN* which encodes the IL-1 receptor antagonist (IL1RA). *IL1RN* was upregulated in the posterior cricoarytenoid muscle 1 week following transection of the recurrent laryngeal nerve in a rat model [58], and IL1RA was significantly increased following 8 h of industrial exposure to respirable and inhalable dust in humans [59]. Together this substantiates a role for the increased *IL1RN* we observed and of a possible active innate immunity repression in response to the low humidity challenge.

Conversely, the upregulation of *MMP12* and *MCP1* genes may suggest the activation of inflammatory macrophages. *MMP12* was the most significantly upregulated gene in this study by RNA-Seq and RT-qPCR. MMP12 exhibits proteolytic activity on multiple ECM components including elastin, fibronectin, entactin, and type IV collagen [60], all of which are expressed within the vocal folds. Although called “macrophage elastase”, it is also expressed in human vocal fold fibroblasts [61] and bronchial epithelial cells in vitro [62], and in both superficial and deep epidermal layers of the skin in response to ultraviolet radiation [63]. MMP12 has a potential role in the development of dysphonia following low humidity exposure since type IV collagen and elastin play an important role in the viscoelasticity and phonatory function of the vocal folds [64, 65]. MMP12 may contribute directly to inflammation though epidermal growth factor receptor (EGFR) dependent induction of IL-8 from the respiratory epithelium [66]. Interestingly, MMP12 has been shown to positively influence wound healing following epithelial injury to the cornea [67], so it is unclear if the upregulated response to low humidity would be deleterious or influence a reparative response in the vocal folds. *MCP1* is an α-defensin expressed in the lungs of fetal and adult rabbits [68]; it is secreted from neutrophils and rabbit lung macrophages and exhibits broad antimicrobial activity In our study, the expression of *MCP1* was novelly detected in the rabbit larynx, and its upregulation in repsose to low humidity warrants further investiation including targeted anaylsis of differential expression between inflammatory cells and the larygeal tissue.

It is not surprising to find evidence of a pro-inflammatory response with surface dehydration as other environmental stressors such as simulated acidic reflux [69], hypertonic challenge [38], and phonotrauma [47, 70] can perturb the epithelial tight junctions of the vocal folds—indicative of the activation of proinflammatory pathways. As we did not investigate for cell-specific gene expression in this study, we are limited to conclude if the upregulation of these genes reflects activation of macrophages or activity of the epithelium or lamina propria fibroblasts, and further study is warranted. An intriguing hypothesis for a case of macrophage activation would be altered response to local microbiome or pathogens resulting from changes to the laryngeal microenvironment following dehydration.

The perturbation of ion transport or other lubrication mechanisms is anticipated as a response to the altered hydration state of the laryngeal surface [71]. Although no gene or protein interaction enrichment cluster was identified within the 103 DEGs analyzed, presumably due to the diversity of substrate and transporter type, a considerable set of ion and solute transporter related genes were identified by RNA-Seq, including *ECCP*, *SLC5A1*, *SLC13A5, SLC23*A1, *SLC27A2*, and *ZACN.* All SLC family members were downregulated. This set represents predominantly ion transport, with SLC13A5 and SLC27A2 being involved in glucose transport and fatty acid ligation. In vitro studies of human nasal epithelial cells [36] and human bronchial cell culture [72] demonstrated that apical osmotic pressure can result in altered epithelial electrolyte transport; however, studies with canine tracheal and bronchial cell culture [73] and an in vivo canine model [74] concluded that not all epithelial fluid flux is coupled to electrolyte transport. This evidence suggests that the epithelium may respond to either aberrant electrolyte concentrations or non-ionic osmotic pressure. It is not surprising to find evidence of altered chloride secretion specifically, as balanced sodium and chloride ion secretion is attributed to volume regulation of the airway surface fluid, but the contribution of transport of other ionic and non-ionic species is not well described for airway surface fluid regulation. Our results suggest the pertinence of future targeted study of noncanonical secretion products in the respiratory tract.

Although the *ECCP* is annotated as an epithelial chloride channel protein, the translation product for *ECCP* is neither well characterized nor has a direct ortholog in humans. It may belong to the calcium-activated chloride channel proteins (CLCA) family as identified by conserved functional domains, although it exhibits limited homology to the rabbit CLCA proteins. The genes for *CLCA1, CLCA2,* and *CLCA4* lie within the same genetic neighborhood as *ECCP* but were identified by RNA-Seq with FDR > 0.99, indicating they are not differentially expressed in our model of surface dehydration (Additional file 1: Table S1). This suggests a distinct role for *ECCP* and its downregulation that warrant further investigation as an ion channel protein newly described in the context laryngeal surface dehydration. In contrast to *ECCP*, ZACN was upregulated in low humidity compared to moderate humidity but failed to reach statistical significance by RT-qPCR. ZACN is a cation channel expressed in the human trachea and other tissues and demonstrates permeability to potassium ions but not to chloride ions [75]; there is no discussion of its expression in the vocal folds in the literature, and it is unclear if it may also be sodium ion permeable. Taken together with the SLC family members identified, these results support a potential role for solute flux as a homeostatic response to surface dehydration. Interestingly, however, the downregulation of chloride transportation would be a counterintuitive response to surface dehydration at the apical membrane as chloride is generally directed out of the cell and aberrant chloride transport can be detrimental in the airways as seen in cystic fibrosis. There is a distinction between the respiratory epithelium of the airways and the nonkeratinized stratified squamous epithelium of the vocal folds, so care must be taken with direct translations of actions between the two.

The mucins are equally important to maintain satisfactory hydration of the laryngeal surface as ion and fluid flux. *MUC12*, *MUC21*, and *TFF1* were identified as downregulated by RNA-Seq. Both mucins are members of the cell-surface associated mucin family, and as such, should originate directly from the epithelial cells. The first exon of *MUC12* exhibited increased expression in laryngeal epithelium from laryngeal reflux patients compared to reflux negative patients [76]. Exogenous surface expression of MUC21 in in vitro cell culture reduced intercellular adhesion and adhesion to extracellular components [77]. It is interesting then to observe all three to be downregulated. However, in addition to roles as epithelial protectants, mucins and related proteins also serve roles in cell signaling with physiological consequences. This is recently shown for MUC21 overexpression as influencing the development of lung adenocarcinoma [78] and TFF1 influencing epithelial-mesenchymal transition. Together, this may be a contributing factor to the STRING cluster of keratinocyte differentiation factors discussed below, but further study is warranted to determine which cell types are expressing these genes and which cell signaling may be impacted.

Although there was no gross inflammation observed, some level of epithelial cellular response to surface dehydration is expected. The vocal folds are covered by a non-keratinized stratified squamous epithelium for which some aspects of development are well understood, such as embryological developmental factors and differentially expressed structural components [79, 80], but a comprehensive molecular description is not available as for other epithelia like the epidermis. It is interesting that several keratinocyte developmental and epithelial structural factors were identified with RNA-Seq, enriched in the low humidity group by GSEA, and as a protein interaction cluster in the STRING analysis: CDSN, CNFN, CRNN, KRT80, KRTDAP, and TGM3. Also identified by RNA-Seq were *SPBN*, another keratinocyte factor, and *CDHR4*, a cell interaction mediator. All of these were downregulated. *SPBN*, *CDHR4*, and *CDSN* were selected for RT-qPCR validation. All three gene products may be involved in maintaining the integrity of the stratified squamous epithelium, though none have been described specifically within the vocal folds until this study. SPBN is expressed in the suprabasal layers of tongue, stomach, and epidermis [81]. It is required for keratinocyte differentiation in an in vitro skin model [82] and skin development in murine embryos [83]. The specific activity of CDHR4 is not described in the literature, but family member CDHR2 is expressed in gastrointestinal epithelial cells and is associated with microvillus development [84], while family member CHDR3 is expressed in ciliated respiratory epithelial cells and is associated with ciliary development and intercellular interactions [85]. CDSN is expressed in the stratum granulosum of human skin and appears to participate in cellular cohesion at this level, with its loss associated with desquamation [86, 87]. That the entire cluster was downregulated substantiates surface dehydration as capable to influence vocal fold epithelial maintenance. Further study is required to elucidate the specific roles of these proteins within the vocal folds, as this epithelium is distinct from the epidermis.

### Limitations

A limitation of designing an environmental chamber as described here was that it precluded the provision of relative humidity lower than 15%. While environmental rooms and chambers are commercially available, they are cost prohibitive and their small size precludes the use of certain animal models, such as rabbits. Another limitation of the study is that we only observed a single time point after low humidity exposure. It has been shown that local response to vocal fold injury is transient and time-dependent [43, 49, 88]. Further studies specifically observing for inflammatory response at multiple times points within a single challenge or within repeated or chronic challenges would be helpful in further characterization of vocal fold biology. Finally, the dissected vocal fold tissue included striated muscle and small amounts of respiratory epithelium immediately above and below the region of the vocal folds. Therefore, genes associated with muscle or respiratory epithelium were not selected for the discussion.

## Conclusions

In this study, we investigated whether surface dehydration induced by low humidity would affect vocal fold biology. We successfully developed an efficient and cost-effective environmental chamber to induce surface dehydration. Humidity was maintained at occupationally relevant levels to observe for physiological impact. Low humidity challenge did not induce systemic dehydration as evaluated with PCV. High-throughput RNA-Seq provided evidence of a biologic response to low humidity challenge, including changes within stimulus-response, lubricative mechanisms, and potential alterations in epithelial maintenance. This study successfully serves as a framework for targeted investigations of molecular response to surface dehydration in the larynx.

## Methods

### Animals

All experiments were conducted in accordance with the guidelines and after approval of the Purdue Animal Care and Use Committee (Protocol # 1606001428). Animals were obtained from Envigo Global (Indianapolis, IN) and acclimatized for at least 1 week. Male New Zealand White rabbits, six to 8 months of age, and approximately 3 Kg were used for all experiments. Each experiment was run with two rabbits at a time randomly assigned to either the low (*n* = 8) or moderate (*n* = 6) humidity group. Samples sizes were selected based on recommendation from the Purdue Bioinformatics Core to ensure ideal minimum samples for statistical validity of RNA-Seq (*n* = 6 from each group). Changes to PCV were examined for all rabbits. RT-qPCR validation was conducted with 13 rabbits (low *n* = 7, moderate *n* = 6); one rabbit was excluded due to poor quality of RNA obtained after repeat extraction. Food and water were withheld during experiments under both humidity conditions. To encourage consistent, baseline hydration, all animals were pre-hydrated with 0.1 M sucrose in water ad libitum for the 2 days preceding the experiment. Animals were euthanized at the end of the experimental exposure with a single 1.0 mL IV dose of Beuthanasia-D Special (Schering Plough Animal Health Corp., Union, NJ).

### Humidity challenge protocol

Eight hour low humidity and moderate humidity exposure were conducted in a specially fabricated environmental chamber. The chamber interior was segmented into three similar compartments, each with dimensions approximately 61 cm × 61 cm × 46 cm (Fig. 1a, b). Two compartments were sealed to limit the influx of room air and were intended for low humidity exposure, whereas the third compartment was left open to room air and was intended for a moderate humidity control. Gated duct caps were included within the wall of the low humidity compartment to allow for titration of room air as necessary.

Low humidity was achieved with a 70-pint commercial dehumidifier (Hisense DH70K1G: Qingdao, China) set to High Continuous attached to the chamber via 4-in. ducting. Moderate humidity exposure was achieved by opening the chamber airspace to room air without conditioning from the dehumidifier. Internal relative humidity and temperature were tracked using a HOBO Data Logger with a 12-bit Temperature/Relative Humidity Smart Sensor (U14–002, S-THB-M002: ONSET, Bourne, MA) at one-minute intervals.

### Blood collection and analysis

Blood was collected in heparinized tubes at the beginning of the 8-h experiment and immediately prior to euthanasia via venipuncture of the lateral ear vein to minimize trauma and distress of collection. Packed cell volume (PCV) was measured manually by visual assessment using a microhematocrit reader card following centrifugation.

### Sample collection and RNA extraction

The larynx and proximal trachea were excised from each animal immediately following euthanasia. The larynx was bisected posteriorly along the sagittal midline and pinned onto wax to expose the laryngeal lumen. Full-thickness soft tissue was microdissected bilaterally at the level of the glottis under magnification with microdissection scissors. Sections approximately 2-3 mm in any dimension collectively representing the vocal fold and surrounding tissue were immediately stored in RNAlater® Stabilization Solution (Invitrogen, Waltham, MA), stored at 4 °C overnight, and at − 80 °C until processing. Total RNA was extracted with the RNeasy Fibrous Tissue Mini Kit following the manufacturer protocol (QIAGEN®, Hilden, Germany).

### RNA sequencing (RNA-Seq)

RNA quality was assessed by RNA Eukaryotic Pico Chip (Agilent Technologies Inc., Santa Clara, CA) and used to construct poly-A derived cDNA libraries with the Universal Plus mRNA-Seq kit (NuGEN Technologies, Inc., Redwood City, CA). High throughput sequencing was completed with an Illumina® NovaSeq™ 6000 Sequencing System (Illumina Inc., San Diego, CA) by 100 million reads, paired, of 150 bases per sample. Differential gene expression analysis was conducted by the Purdue Bioinformatics Core using Cuffdiff with default parameters [89]. Data were submitted to the NCBI GEO database under accession number GSE148588.

### Quality control and read mapping

Sequence quality was assessed using FastQC (v0.11.7) (https://www.bioinformatics.babraham.ac.uk/projects/fastqc/) for all samples, and quality trimming was done using FASTX-Toolkit (v 0.0.14) (http://hannonlab.cshl.edu/fastx_toolkit/) to remove bases with Phred33 score of less than 30, while retaining the resulting reads of at least 50 bases in length. The quality trimmed reads were mapped against the reference genome of *Oryctolagus cuniculus* using STAR (v 2.5.4b) [90].

### Differential gene expression analysis and annotation

Differential gene expression analysis between low and moderate-humidity groups was carried out using ‘R’ (v 3.5.1; http://www.r-project.org/). STAR mapping (bam) files were used for analysis by the Cuffdiff from Cufflinks (v 2.2.1) [89] suite of programs that perform differential expression analysis based on FPKM values. Syntax is provided in Additional file 4. Cuffdiff uses bam files to calculate Fragments per Kilobase of exon per Million fragments mapped (FPKM) values, from which differential gene expression between the pairwise comparisons can be ascertained. FPKM obtained were used for principal component analysis comparing individual rabbits; low expression genes were not removed. The gene annotations were retrieved from BioMart databases using biomartr package in ‘R’.

### Functional enrichment analysis and predicted protein interactions

Differential gene expression data were filtered for a false discovery rate (FDR) of less than or equal to 0.05. Log2 FC positive values imply upregulation, while negative values imply downregulation of genes in the vocal folds exposed to low humidity versus moderate humidity challenge. The set of differentially expressed genes provided by Cufflinks meeting the FDR criterion (*n* = 103) was used as input by Ensembl gene ID for DAVID (v6.8) [91] to obtain functional annotation analysis with 86 being found within the database. The same set of genes was used as input for STRING (Search Tool for the Retrieval of Interacting Genes/Proteins) v.11.0 (https://string-db.org) for prediction of protein interaction analysis, providing a full, supplemented network of 100 nodes. In parallel, genes were ranked in descending order based on -log10 *P*-value multiplied by the sign of log2 transformed FC as input for GSEAPreranked (versions 7.2.0) that was used to perform gene set enrichment analysis using GO gene sets.

GO and KEGG enrichments were obtained from DAVID with default settings. STRING analysis parameters were set with line thickness representing the strength of the data to support interaction including text mining, experimental, database, co-expression, neighborhood, gene fusion, and co-occurrence sources, the minimum required interaction score set to 0.4, shell parameters set to “None”, and disconnected nodes to be hidden. Clusters were generated based on the Markov Cluster Algorithm with the inflation parameter set to 2. The parameters used and full datasets from GSEA are provided in Additional file 4.

### Quantitative reverse transcription PCR (RT-qPCR)

Total RNA was used to generate cDNA with SuperScript™ IV VILO™ Master Mix (Invitrogen) using 374 ng of RNA as the template. RT-qPCR was performed in triplicate using SYBR Green 2x PCR Master Mix (Applied Biosystems, Waltham, MA) with 0.1 M of each primer and 2.5 μL of template cDNA in a 25 μL reaction volume using a QuantStudio 3 System (Applied Biosystems) thermocycler. Data was collected over 40 cycles by QuantStudio Design & Analysis Software v1.5.1. Primers used in this study are listed in Additional file 3: Table S3. Relative expression quantification of each gene was calculated using the 2^(−ΔΔCt)^ method [92].

### Statistical analysis

Statistical analysis was completed and visualized using RStudio™ Version 1.2.1335 (RStudio Inc., Boston, MA) with libraries *Tidyverse* [93] and *outliers* [94]. Changes in PCV were evaluated with Mann-Whitney nonparametric test. Relative gene expressions from RT-qPCR were tested with Welch two-sample t-test following removal of outlier values as determined by Grubb’s test. A *p*-value < 0.05 was considered statistically significant for all analyses.

## Supplementary Information

**Additional file 1: Table S1.** Complete list of genes identified by RNA-Seq analysis and differentially expressed genes (DEG) identified in the larynges of low humidity exposure group by Cufflinks. Excel table of all DEGs obtained by Cufflinks analysis, including Ensembl gene IDs, statistics, and Biomart annotations. Color code Ensembl_ID: green (FDR < 0.05), yellow (0.05 < FDR ≤ 0.1), red (0.1 < FDR ≤ 0.2).

**Additional file 2: Table S2.** Functional enrichment analyses of differentially expressed genes identified in the larynges of low humidity exposure group. Excel table containing the functional annotation enrichments divided into GO categories and KEGG obtained with DAVID (first tab), the functional annotation enrichments obtained by STRING (second tab), and the enrichement data for moderate and low humidity groups by GSEA.

**Additional file 3: Table S3.** List of primers used in the RT-qPCR validation.

**Additional file 4 Cuffdiff Cmd.** Syntax used for Cufflinks analysis.

## Data Availability

The RNA Sequencing dataset supporting the conclusions of this article are available in the NCBI GEO database under accession number GSE148588 (https://www.ncbi.nlm.nih.gov/geo/query/acc.cgi?acc=GSE148588). Datasets including a full list of genes identified and Cufflinks-calculated differential expression, the functional enrichment analyses of differentially expressed genes, a list of primers used in the RT-qPCR validation, the full GSEA data set, and the syntax used for Cufflinks analysis are provided in Additional files 1, 2, 3 and 4, respectively.

## Abbreviations

CDHR4: Cadherin related family member 4
CDSN: Corneodesmosin
ECCP: Epithelial chloride channel protein
DAVID: Database for Annotation Visualization and Integrated Discovery v6.8
DEGs: Differentially expressed genes
FC: Linear fold change
GO: Gene ontology
KEGG: Kyoto Encyclopedia of Genes and Genomes
MCP1: Macrophage cationic peptide 1
MMP12: Matrixmetalloprotease 12
MUC21: Mucin-21
PCV: Packed cell volume
RT-qPCR: Reverse transcriptase quantitative polymerase chain reaction
SPBN: Suprabasin
STRING: Search Tool for Recurring Instances of Neighboring Genes
ZACN: Zinc activated chloride channel
