# Supplementary material for: RNA sequencing identifies transcriptional changes in the rabbit larynx in response to low humidity challenge
Source: BMC Genomics. 2020 Dec 11;21:888. doi: 10.1186/s12864-020-07301-7 (PMC7733274; doi:10.1186/s12864-020-07301-7)
Supplement: Supplementary file 3 — Additional file 3: Table S3. List of primers used in the RT-qPCR validation. [file 12864_2020_7301_MOESM3_ESM.pdf]

GENE	RT-qPCR Primers (5' → 3')
ECCP	F: TATGCACGAGTCAGCCAAGG<br>R: TCAGCACCTGCCCCATTATC
CDHR4	F: GGTGACACCCGTCAATGAGT<br>R: GAACCTCTCCTGAGACGTAGT
CDSN	F: TCTCCTCCTGCCAGGAACCT<br>R: CTAGAGCTGCTGGAGCCACT
MCP1	F: GCACGTTTCAGTGAGCATCG<br>R: ACCACACCTGCCTTTACACC
MMP12	F: AGGCCATAATGTTTCCCACCT<br>R: CTGCTCTGGGCCTCCATAAAG
MUC21	F: TTCTGTGTGAGAAAGTGCCTGT<br>R: GTGCCCCATCCATCTCCAAG
SPBN	F: GCTGAATGGTGGTCAAGGCG<br>R: ATGTTGGCGACGTTCCTCCA
ZACN	F: AACTGCGACTTTGAGCTCCT<br>R: TGACCACGTATTCCCGCTTG
